# Multi-omics joint analysis reveals how *Streptomyces albidoflavus* OsiLf-2 assists *Camellia oleifera* to resist drought stress and improve fruit quality

**DOI:** 10.3389/fmicb.2023.1152632

**Published:** 2023-03-17

**Authors:** Zhilong He, Kunpeng Cui, Rui Wang, Ting Xu, Zhen Zhang, Xiangnan Wang, Yongzhong Chen, Yonghua Zhu

**Affiliations:** ^1^Research Institute of Oil Tea Camellia, Hunan Academy of Forestry, Changsha, China; ^2^National Engineering Research Center for Oil Tea Camellia, Changsha, China; ^3^Hunan Province Key Laboratory of Plant Functional Genomics and Developmental Regulation, College of Biology, Hunan University, Changsha, China

**Keywords:** *Camellia oleifera*, endophyte, drought stress, microbiomes, transcriptomes, metabolomes

## Abstract

*Camellia oleifera* (*C. oleifera*) is a unique edible oil crop in China cultivated in the hilly southern mountains. Although *C. oleifera* is classified as a drought-tolerant tree species, drought remains the main factor limiting the growth of *C. oleifera* in summer and autumn. Using endophytes to improve crop drought tolerance is one effective strategy to meet our growing food crop demand. In this study, we showed that endophyte *Streptomyces albidoflavus* OsiLf-2 could mitigate the negative impact of drought stress on *C. oleifera*, thus improving seed, oil, and fruit quality. Microbiome analysis revealed that OsiLf-2 treatment significantly affected the microbial community structure in the rhizosphere soil of *C. oleifera*, decreasing both the diversity and abundance of the soil microbe. Likewise, transcriptome and metabolome analyses found that OsiLf-2 protected plant cells from drought stress by reducing root cell water loss and synthesizing osmoregulatory substances, polysaccharides, and sugar alcohols in roots. Moreover, we observed that OsiLf-2 could induce the host to resist drought stress by increasing its peroxidase activity and synthesizing antioxidants such as cysteine. A multi-omics joint analysis of microbiomes, transcriptomes, and metabolomes revealed OsiLf-2 assists *C. oleifera* in resisting drought stress. This study provides theoretical and technical support for future research on endophytes application to enhance the drought resistance, yield, and quality of *C. oleifera*.

## 1. Introduction

*Camellia oleifera* is a unique edible oil tree species widely distributed in South China, particularly in Hunan, Jiangxi, and Guangxi (Xu et al., 2021). They contain saponins, polyphenols, and flavonoids which are rich secondary metabolites that have garnered attention (Yu et al., 2018). Camellia oil contains more than 90% unsaturated fatty acids, enhances human immunity, lowers blood pressure, and prevents cardiovascular and cerebrovascular diseases. As a result, it is called “Oriental olive oil” and has broad market development prospects (Cheng et al., 2014).

Drought is a severe problem opposing agricultural development worldwide. *C. oleifera*, planted in the hilly southern mountains, is a drought-tolerant tree species. Even so, drought is the main obstacle limiting yield in summer and autumn (He et al., 2022). Drought can adversely affect plant growth through wilting, weakening photosynthesis, reducing fruit oil content, and decreasing flower bud numbers and fruit set rate, potentially eliminating drought-sensitive species (Dong et al., 2017). Moreover, drought stress can significantly inhibit fruit cell expansion. For example, if *C. oleifera* is subjected to drought stress during the rapid fruit cell expansion period, the final fruit volume can only reach 85% of the average (Li et al., 2015). This decrease was also seen in citrus and peach trees (Mercier et al., 2009; García-Tejero et al., 2010). Additional studies have focused on cultivation, drought-resistant screening, photosynthesis, and physiological and biochemical responses (Wang et al., 2015; Qu et al., 2019; He et al., 2020). Thus, a solid foundation has been laid for improving the drought tolerance of *C. oleifera*.

While plants adapt to water-scarce environments by altering their morphology and physiology, they sometimes require associated microbiome support to alleviate burdens from stressful conditions. In recent years, increasing attention has centered on synergistically using beneficial microorganisms for plant defense against abiotic stresses, improving drought resistance. Various microorganisms have drought tolerance advantages that can help host plants cope with drought stress. Many studies have proven this strategy eco-friendly, cost-effective, sustainable, and effective (Olenska et al., 2020). This was demonstrated when isolating drought-tolerant *Bacillus* and *Bacillus pseudoocheri* from wheat and white grass rhizospheres, respectively (Chakraborty et al., 2013). Ali et al. (2013) isolated and screened several *Pseudomonas* strains with drought tolerance and growth-promoting properties from different crops’ rhizosphere-and non-rhizosphere soils. Shirinbayan et al. (2019) obtained drought-tolerant *Azotobacter* strains from rhizosphere soil samples collected from pastures and crops grown in semi-arid regions of Iran. Several drought-tolerant bacteria, such as *Acinetobacter calcoaceticus*, *Penicillium* sp., *Pseudomonas libanensis*, and *Streptomyces laurentii*, have also been isolated from different grain rhizospheres (Kour et al., 2020; Kour and Yadav, 2022).

Compared with rhizosphere soil microorganisms, endophytes that live symbiotically in internal plant tissues positively impact plant physiology more (Dutta et al., 2014). Among the beneficial endophytes, actinobacteria, especially *Streptomyces*, are high-quality resource pools with numerous secondary metabolites (Cui et al., 2022). Some endophytic *Streptomyces* has reportedly induced systemic tolerance to abiotic stressors in plants. In drought conditions, drought-tolerant endophytic *S. coelicolor* DE07, *S. olivaceus* DE10, and *S. geysiriensis* DE27 could promote wheat growth and increase yield by producing phytohormones both in greenhouse and field (Yandigeri et al., 2012). Furthermore, Endophyte B26 improved *Phleum pratense* L. growth by altering osmolyte accumulation in roots and shoots (Gagne-Bourque et al., 2016). To date, there are only a few reports on endophytic actinobacteria promoting phosphorus nutrient uptake and growth in *C. oleifera* (Cui et al., 2022). In comparison, there are even fewer reports on using endophytic *Streptomyces* to assist *C. oleifera* in resisting drought stress.

Previously, we isolated an endophytic *Streptomyces albidoflavus* OsiLf-2 which could improve host rice resistance to drought and salt stresses (Niu et al., 2022). Therefore, we hypothesized that OsiLf-2 would have the same effect on woody oil crops. This study aims to determine the effect and mechanism of OsiLf-2 on improving *C. oleifera* drought resistance and fruit quality through a multi-omics analysis of microbiomes, transcriptomes, and metabolomes.

## 2. Materials and methods

### 2.1. Plant material and treatment

The seven-year-old *Camellia oleifera* ‘Xianglin No. 1’ used in this study was planted in an artificially arranged experimental field at the Camellia Germplasm Resource Bank of Oil-tea Camellia Research Institute, Hunan Academy of Forestry Sciences located at 113°01′E, 28°06′N, and 80–100 m above sea level. It belongs to a subtropical monsoon climate, with an annual average temperature of 16.8–17.3°C, annual average rainfall of 1,422 mm, a frost-free period of 275 days, and annual average relative humidity of 80%. The soil classifies as Quaternary red soil with a pH between 4.5 and 5.5, an organic matter content of 41.01 g·kg^−1^, a total nitrogen content of 2.68 g·kg^−1^, a total phosphorus content of 0.61 g·kg^−1^, and a total potassium content of 4.53 g·kg^−1^. The planting density was 4 by 3 m. Organic fertilizer had been applied once a year in December and 3 kg of organic fertilizer was added to each mature tree (>5-year-old) per year. The method of furrow application was adopted, and the fertilizing ditch was outside the projection line of the tree crown. This study included blank control, drought control, and drought plus endophyte experimental groups. In the drought treatment group, soil mulching and tree shading were carried out for each tree to control the soil moisture content to an extremely low level from July to October 2021. In contrast, the control group was irrigated daily to the standard field capacity. Ten biological replicates per treatment group were tested.

*Streptomyces albidoflavus* OsiLf-2 (GenBank accession number: NZ_MNPQ00000000.1, China Microorganism Collection Center accession number: CGMCC 11673) is an endophytic actinobacteria isolated from rice leaves (Gao et al., 2019). OsiLf-2 was cultivated in a Mannitol-soybean (MS) medium at 30°C for approximately 5 days. Mature spores were then scraped off with a sterile cotton swab and dissolved in sterile water to prepare a spore suspension. The concentration of this spore suspension was adjusted to 1 × 10^8^ spores mL^−1^ on a hemocytometer (Niu et al., 2022). Next, each *C. oleifera* was inoculated with 1 L of the prepared spore suspension through root irrigation and then subjected to drought stress after one month.

### 2.2. Determination of germplasm and *Camellia oleifera* fruit oil quality traits

Fruits from each treatment group were harvested during *C. oleifera’s* fruiting stage. A vernier caliper was used to measure fruit height, diameter, peel thickness (top, middle, and base), and seed diameter and height, fruits and seeds were also weighed (Yang et al., 2022). Ten fruits per tree were picked. Oil content and palmitic, stearic, oleic, linoleic, and linolenic acids were used as oil quality indicators. They were determined according to the Chinese Standard (GB-5009, 168–2016) (You et al., 2019). The assay was performed in triplicate.

### 2.3. Microbiome analysis on *Camellia oleifera’s* rhizosphere soil

The rhizosphere soil samples from *C. oleifera* roots in the drought (Control group) and drought with OsiLf-2 (LF group) treatment groups were quick-frozen with liquid nitrogen and stored at-80°C. Genomic DNA was extracted from the soil using a PowerSoil DNA Isolation kit (MoBio Laboratories, Inc., Carlsbad, CA, United States, Catalog: 12888–50-1), and its purity and quality were assessed on 1.0% agarose gel. Primers 338F (ACTCCTACGGGAGGCAGCAG) and 806R (GGACTACH VGGGTWTCTAAT) were used to amplify the bacterial 16S rRNA gene’s V3-4 hypervariable region (Munyaka et al., 2015). PCR products were purified using the Agencourt AMPure XP Nucleic Acid Purification kit (Beckman Coulter, Inc., Indianapolis, United States, Catalog: A63881) and quantified with real-time PCR. High-throughput sequencing was performed using Illumina Miseq PE250 at Beijing Allwegene Gene Technology Co., Ltd., and optimized sequences were obtained by sequence assembly, filtration, and chimera removal. Subsequently, OTU clustering and annotation analyses were performed (Edgar, 2013). Alpha diversity analyses were used QIIME (v1.8.0) (Miller et al., 2016) to generate rarefaction curves and to calculate the richness and diversity indices based on the OTU information, and R (v3.6.0) software was used to plot (Zhou et al., 2020). To describe the dissimilarity between multiple samples, the Beta Diversity distance matrix between samples was calculated using the Bray Curtis algorithms, and the PCA analysis plot was performed using R (v3.6.0) software based on the distance matrix (Wang et al., 2012). Annotation analysis results can provide taxonomic information at each level, enabling analysis of sample composition and differences in the community structure between samples.

### 2.4. *Camellia oleifera* root transcriptome analysis

*Camellia oleifera* root tissues were collected from the Control and LF groups, and three biological replicates were performed for each treatment group. Total RNA was extracted by TRIzol (Invitrogen, CA, United States), and RNA quality was assessed. Next, the RNA samples were subjected to library construction and sequencing in Beijing Allwegene Gene Technology Co., Ltd. using the Illumina Novaseq 6,000, PE 150 sequencing platform. STAR software aligned the clean data to the reference genome (*Camellia sinensis* genome GCF_004153795.1) (Dobin et al., 2013). Each sample’s gene expression level and differential expression were analyzed using HTSeq and DESeq software, respectively (Anders and Huber, 2010; Anders et al., 2015). | log_2_ (Fold Change) |>1 and the *q* value <0.05 were used as the cutoff criteria to screen the differentially expressed genes (DEGs) between LF and Control groups. The GOseq R package was used for the gene ontology (GO) enrichment analysis of the DEGs (Young et al., 2010), and KOBAS software was used to analyze DEG enrichment in KEGG pathways (Mao et al., 2005).

### 2.5. *Camellia oleifera* root non-target metabolome analysis

*Camellia oleifera* root tissues were collected from the Control and LF groups, and six biological replicates were performed for each. The tissue samples were subjected to metabolite extraction. Fifty mg of samples were homogenized with 500 μL pre-cooled methanol: water (3:1) at 35 Hz for 4 min, then ultrasonic treated with ice water bath for 5 min. After centrifugation at 12,000 rpm at 4°C for 15 min, 200 μL supernatant was evaporated in a vacuum concentrator, and 40 μL of Methoxyamination hydrochloride (20 mg/mL in pyridine) was added and then incubated at 80°C for 30 min, then derivatized by 60 μL of BSTFA regent (1% TMCS, *v*/*v*) at 70°C for 1.5 h. After gradually cooling to the room temperature, 5 μL of FAMEs (in chloroform) was added. Metabolomics data analysis was performed using gas chromatography quadrupole time of flight mass spectrometry technology (GC-QTOF-MS, Agilent 7,890) equipped with a DB-5MS capillary column (30 m × 250 μm × 0.25 μm, J&W Scientific, Folsom, CA, United States) (Garcia and Barbas, 2011; Tsugawa et al., 2011). Further analysis of metabolites was assisted by Beijing Allwegene Gene Technology Co., Ltd. The relationship between metabolite expression and sample category was modeled using orthogonal partial least squares discriminant analysis (OPLS-DA) regression to achieve sample category modeling predictions (Boccard and Rutledge, 2013). GC-QTOF-MS metabolomic data were analyzed through multivariate statistical methods. Card value standards were set for screening differential metabolites: the Student’s *t*-test *p*-value was less than 0.05, and the variable importance of projection in the OPLS-DA model’s first principal component was greater than 1. Subsequently, differential metabolites were projected to authoritative metabolite databases, such as KEGG^1^ and MetaboAnalyst,^2^ to identify involved pathways. Further metabolic pathway analysis on the differential metabolites examined the correlation between metabolic pathways and biological issues.

### 2.6. Data analysis

SPSS (v22.0) (Chicago, IL, United States) was used to analyze experimental data statistically, and Duncan’s multiple-range test was used for ANOVA. Different lowercase letters indicate significant differences at *P* < 0.05.

## 3. Results

### 3.1. Effects of various treatments on *Camellia oleifera* seeds and oil quality traits

While drought stress can directly affect *C. oleifera* fruit development and reduce its yield, beneficial microorganisms-endophytes can improve plant resistance to abiotic stresses. To investigate whether OsiLf-2 alleviates *C. oleifera’s* drought-induced yield reduction under drought stress, OsiLf-2 was applied to the *C. oleifera* planted in the experimental field, and plants were then subjected to drought treatment. As shown in Table 1, compared with the blank control group (CK), peel thickness and fruit height, diameter, and weight were significantly reduced under drought conditions. Meanwhile, drought adversely affected seed height (reduced by 5.63%), diameter, and weight (reduced by 10.68%). OsiLf-2 treatment significantly alleviated the adverse effects of drought stress on *C. oleifera* yield. Furthermore, OsiLf-2-treated fruits and seeds under drought stress were superior to the drought control group and almost reached the same level as the CK group. These results indicated that exogenous OsiLf-2 supplication mitigates the adverse effects of drought stress on the fruit and seed quality of *C. oleifera*.

**Table 1:** Effects of different treatment groups on seed traits of *Camellia oleifera.*

	Fruit traits						Seed traits		
	Fruit height/mm	Fruit diameter/mm	Fruit weight/g	Peel thickness – top/mm	Peel thickness – middle/mm	Peel thickness – bottom/mm	Seed height/mm	Seed diameter/mm	Seed weight/g
CK	50.77 ± 0.76a	43.24 ± 0.47a	49.21 ± 1.54a	10.85 ± 0.31ab	4.75 ± 0.16a	4.70 ± 0.26a	24.13 ± 1.29a	15.82 ± 0.51a	3.09 ± 0.90a
Drought	46.11 ± 1.00b	38.59 ± 1.14b	32.18 ± 1.73b	11.65 ± 0.45a	4.71 ± 0.17a	4.53 ± 0.12a	22.77 ± 0.45a	15.79 ± 0.63a	2.76 ± 0.27a
Drought + OsiLf-2	47.76 ± 0.71b	42.63 ± 0.92a	45.71 ± 2.37a	10.07 ± 0.27b	4.70 ± 0.09a	4.50 ± 0.10a	23.18 ± 0.10a	15.18 ± 0.37a	3.01 ± 0.19a

CK, *Camellia oleifera* without drought treated; Drought, Drought-treated *Camellia oleifera*, Drought + OsiLf-2, *Camellia oleifera* inoculated with OsiLf-2 and treated with drought. Duncan ‘s multiple-range test was used to analyze the significant difference between different letters (ANOVA, *P* < 0.05). Data were expressed as mean ± SE (*n* = 10).

Oil quality is a significant factor that affects fruit quality. Drought stress decreases the content of oil and stearic, oleic, and linolenic acids in *C. oleifera* fruits (Table 2). Specifically, compared to the CK group, the drought control group’s oil content was significantly reduced by 26.09%, and linolenic acid content was decreased by 24.14%. On the contrary, OsiLf-2 application to *C. oleifera* under drought conditions significantly increased fruit oil content. Compared with the CK group, the OsiLf-2 group had a slightly increased in oil content, and the linolenic acid content increased by 54.02%. Moreover, we observed that drought stress only group, and OsiLf-2 application under drought stress group increased fruit palmitic and linoleic acid content by 57.71% and 78.95%, respectively. This indicated that OsiLf-2 application helps improve the fruit oil quality index of *C. oleifera* under drought stress, thus promoting fruit quality.

**Table 2:** Effects of different treatment groups on oil quality traits of *Camellia oleifera* fruit.

	Palmitic acid/%	Stearic acid/%	Oleic acid/%	Linoleic acid/%	Linolenic acid/%	Oil content in kernel/%
CK	7.02 ± 0.11b	2.38 ± 0.02a	83.83 ± 0.07a	5.51 ± 0.06b	0.87 ± 0.05ab	0.46 ± 0.003a
Drought	8.58 ± 0.26a	1.77 ± 0.08b	79.59 ± 1.66b	8.69 ± 1.32a	0.66 ± 0.16b	0.34 ± 0.003b
Drought + OsiLf-2	7.90 ± 0.37ab	1.89 ± 0.05b	78.48 ± 0.37b	9.86 ± 0.32a	1.34 ± 0.25a	0.47 ± 0.006a

CK, *Camellia oleifera* without drought treated; Drought, drought-treated *Camellia oleifera*; Drought + OsiLf-2, *Camellia oleifera* inoculated with OsiLf-2 and treated with drought. Duncan’s multiple-range test was used to analyze the significant difference between different letters (ANOVA, *P* < 0.05). Data were expressed as mean ± SE (*n* = 10).

### 3.2. Response of rhizosphere soil bacterial community to drought stress

**Figure 1:**
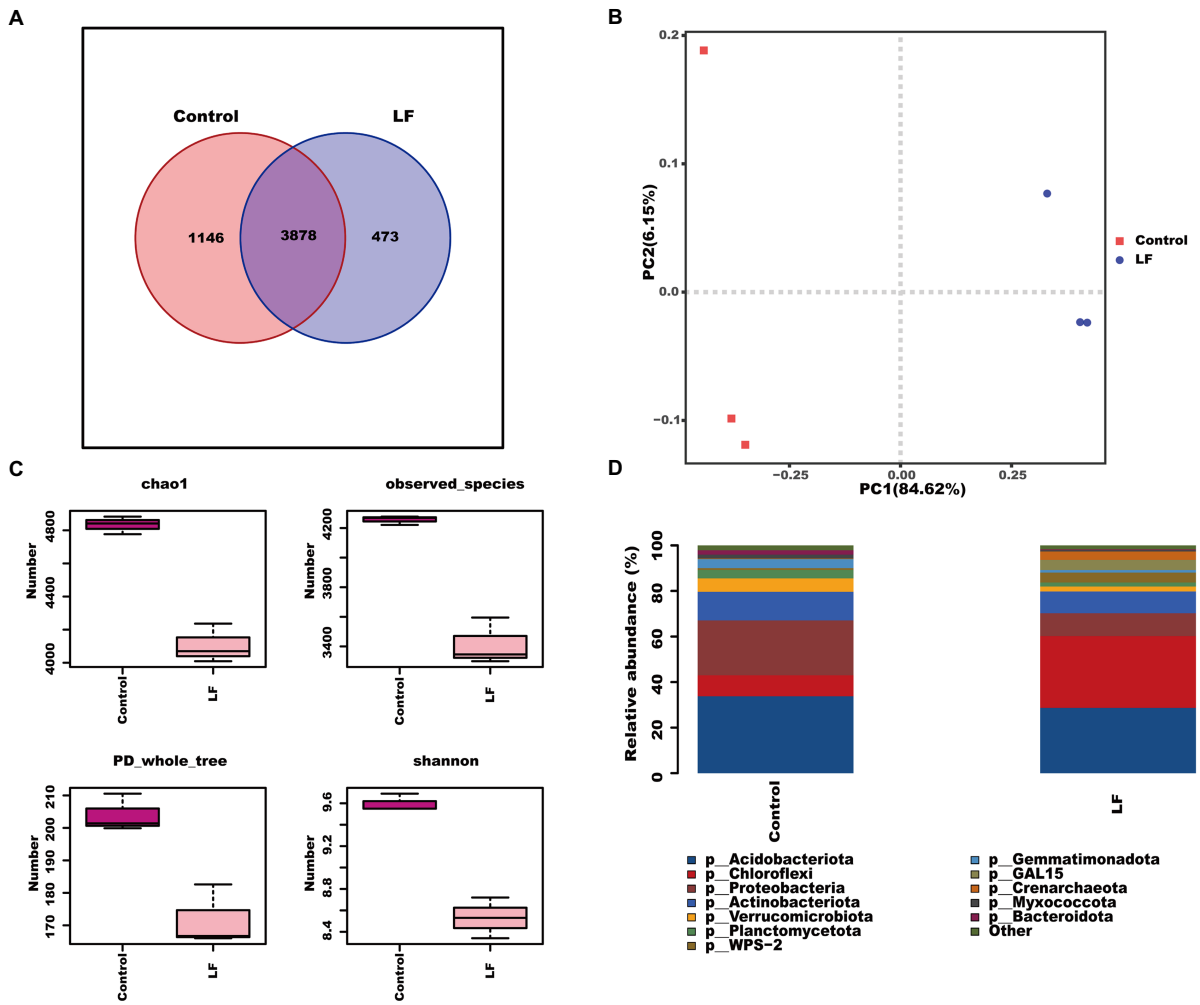
Response of rhizosphere soil biodiversity of *Camellia oleifera* to drought stress. **(A)** OTUs distribution Venn diagram; **(B)** PCA principal component analysis between samples; **(C)** alpha diversity index; **(D)** analysis of microbial composition at the level of phylum.

We further sequenced and analyzed the rhizosphere soil microbial diversity to understand the effect of OsiLf-2 on the rhizosphere bacterial community of *C. oleifera*. The Venn diagram of OTUs distribution showed that the control group and LF group shared 3,878 OTUs, the control group had 1,146 unique OTUs, and the LF group had 473 unique OTUs (Figure 1A). Further PCA analysis revealed a marked separation between control and LF sample groups, indicating differences in the microbial community structure between the two sample groups, with PC1 affecting 84.62% of bacterial communities (Figure 1B). Alpha diversity analysis showed that the rhizosphere soil bacterial diversity index Chao1, PD whole tree, and Shannon were lower in the LF group than in the Control group, indicating that the soil bacterial community’s abundance and diversity decreased after OsiLf-2 addition comparatively (Figure 1C). The relative abundance analysis found that, at phylum levels, the relative abundances of most microorganisms in the LF group were lower than in the Control group (Figure 1D). In contrast, the relative abundance of Chloroflexi, WPS-2, GAL15, and Crenarchaeota in the LF group were higher than in the control group. These results were in agreement with those of our Alpha diversity analysis.

### 3.3. *Camellia oleifera* root transcriptome analysis under drought stress

The six samples in the Control and LF groups were sequenced, and a total of 258,801,714 raw reads were sequenced, and after quality control, 253,176,276 clean reads were obtained, totaling 37.95G Clean Data. The raw data has been uploaded to the Sequence Read Archive as a BioProject (PRJNA935928). The Clean Data of each sample reached more than 5.8 Gb, and the error rates of the six samples were less than or equal to 0.03%, Q20 was greater than 97%, Q30 was greater than 94%, and GC content ranged from 45.23 to 46.09% (Supplementary Table S1), which indicated that the sequencing data was valid. The analysis of the obtained reads against the reference genome revealed that the number of sequences that could be localized to the reference genome was higher than 86% in all samples, and the percentage of reads that were compared to multiple positions was about 10% of the total (Supplementary Table S2). These indicated that the transcriptome data are highly matched and can be used for subsequent gene-level analysis.

|log_2_ (Fold Change)|>1 and the *q* value <0.05 were used as the criteria to screen for DEGs between the LF and Control groups. As shown in Figure 2A 1,445 DEGs were identified from all sequenced samples, including 703 up-regulated and 742 down-regulated DEGs. GO enrichment analysis revealed that among the 1,445 DEGs, 122 up-regulated and 166 down-regulated DEGs were annotated into three categories: biological process (BP), cellular component (CM), and molecular function (MF) (Figures 2B,C).

**Figure 2:**
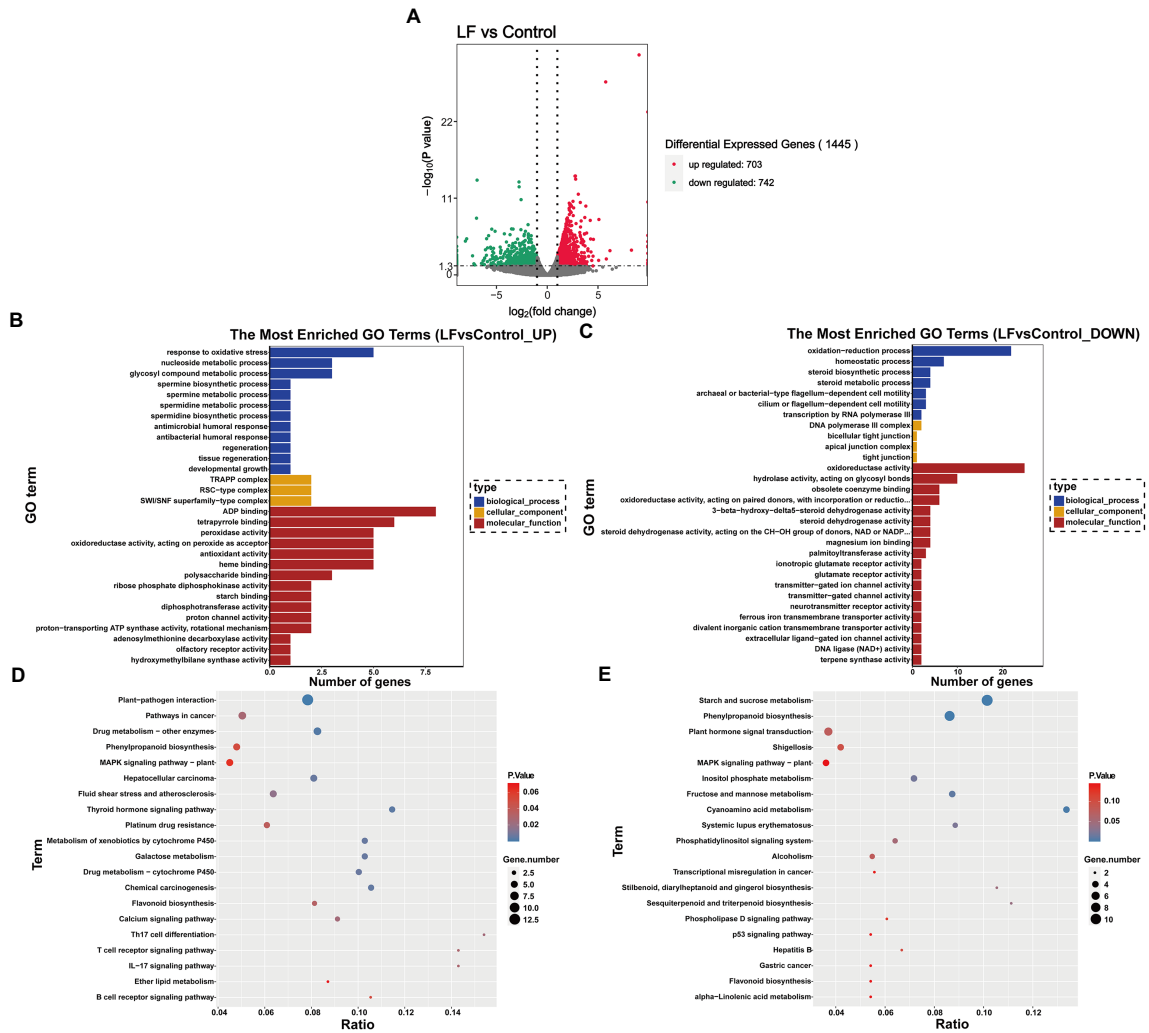
Functional enrichment analysis of differentially expressed genes. **(A)** Differential gene volcano map; **(B)** GO enrichment histogram of up-regulated differential genes; **(C)** GO enrichment histogram of down-regulated differential genes; **(D)** KEGG enrichment scatter diagram of up-regulated differential genes; **(E)** KEGG enrichment scatter plot of down-regulated differential genes.

Among the GO terms related to up-regulated differential genes, 5 up-regulated differential genes (Novel13433, Novel13432, Novel11181, Novel02124, Novel11024) were annotated into the response to oxidative stress in BP term, peroxidase activity, oxidoreductase activity, acting on peroxide as acceptor and antioxidant activity in MF term; 3 up-regulated differential genes (Novel06804, Novel06807, Novel06560) were annotated into glycosyl compound metabolic process in BP term; 3 up-regulated differential genes (Novel11368, Novel11367, Novel03250) were annotated into polysaccharide binding in MF term; 2 up-regulated differential genes (Novel11367, Novel11368) were annotated into starch binding in MF term (Figure 2B).

Among the GO terms related to down-regulated differential genes, 22 down-regulated differential genes were annotated into oxidation–reduction process in BP term; 4 down-regulated differential genes (Novel00528, Novel12914, Novel13348, Novel13346) were annotated into steroid biosynthetic process and steroid metabolic process in BP term, and steroid dehydrogenase activity in MF term; 25 down-regulated differential genes were annotated into oxidoreductase activity in MF term; 2 down-regulated differential genes (Novel03343, Novel03344) were annotated into glutamate receptor activity and ionotropic glutamate receptor activity in MF term (Figure 2C) .

In the KEGG pathway-enrichment analysis, matches were found for 89 up-regulated DEGs and 84 down-regulated DEGs which were mapped to KEGG pathways. Among the up-regulated DEGs, 5 up-regulated DEGs (genemark-HiC_scaffold_7-processed-gene-1604.8, maker-HiC_scaffold_5-snap-gene-831.9, maker-HiC_scaffold_5-snap-gene-833.0, Novel02124, Novel11024, Novel13433) were annotated into Phenylpropanoid biosynthesis pathway; 3 up-regulated DEGs (augustus_masked-HiC_scaffold_7-processed-gene-1085.5, genemark-HiC_scaffold_7-processed-gene-1604.8, Novel08117) were annotated into Flavonoid biosynthesis pathway; 13 up-regulated DEGs were annotated into Plant-pathogen interaction (Figure 2D). For the down-regulated DEGs, 11 down-regulated DEGs were annotated into Starch and sucrose metabolism pathway; 9 down-regulated DEGs were annotated into Phenylpropanoid biosynthesis pathway; 6 down-regulated DEGs (augustus_masked-HiC_scaffold_11-processed-gene-1778.4, augustus_masked-HiC_scaffold_12-processed-gene-2013.7, maker-HiC_scaffold_12-snap-gene-508.10, maker-HiC_scaffold_2-snap-gene-298.5, Novel03722, Novel05187) were annotated into Plant hormone signal transduction pathway; 2 down-regulated DEGs (Novel02307, Novel04836) were annotated into Sesquiterpenoid and triterpenoid biosynthesis; 2 down-regulated DEGs (augustus_masked-HiC_scaffold_7-processed-gene-1891.11, snap_masked-HiC_scaffold_12-processed-gene-1939.19) were annotated into Flavonoid biosynthesis; 4 down-regulated DEGs (maker-HiC_scaffold_2-snap-gene-1005.17, Novel05490, Novel09119, Novel11114) were annotated into Fructose and mannose metabolism; 4 down-regulated DEGs (maker-HiC_scaffold_6-snap-gene-1102.25, maker-HiC_scaffold_8-snap-gene-1144.58, Novel03454, Novel03746) were annotated into Cyanoamino acid metabolism (Figure 2E).

The above GO and KEGG pathway analysis showed that the mentioned DEGs are involved in multiple pathways, such as antioxidant and oxidoreductase activity, oxidative stress response, steroid biosynthesis and metabolism, phytohormone signaling, sugar, and sugar alcohol synthesis pathways, which are closely related to plant physiological and biochemical responses to drought stress (Figures 2B–E). These findings suggest that OsiLf-2 induces host plants to activate their antioxidant defense system and enhance antioxidant capacity. In addition, this promotes flavonoid synthesis and osmolyte production, such as sugar alcohols and polysaccharides, to increase cellular osmotic pressure and improve host drought resistance.

### 3.4. *Camellia oleifera* root metabolome analysis under drought stress

#### 3.4.1. Mass spectrometry data analysis and statistics

Next, we performed non-target metabolomics analyses on 12 paired root samples from LF and Control groups. Then, mass spectrometry data were subjected to missing value padding and low-mass ion removal (removing ions for which more than 50% of the masses were missing in QC samples or more than 80% of the masses were missing in actual samples), followed by data filtering. Finally, statistical ion information was collected. As shown in Supplementary Table S3, 245 ions were obtained, and 226 (92.24%) remained after pretreatment.

**Figure 3:**
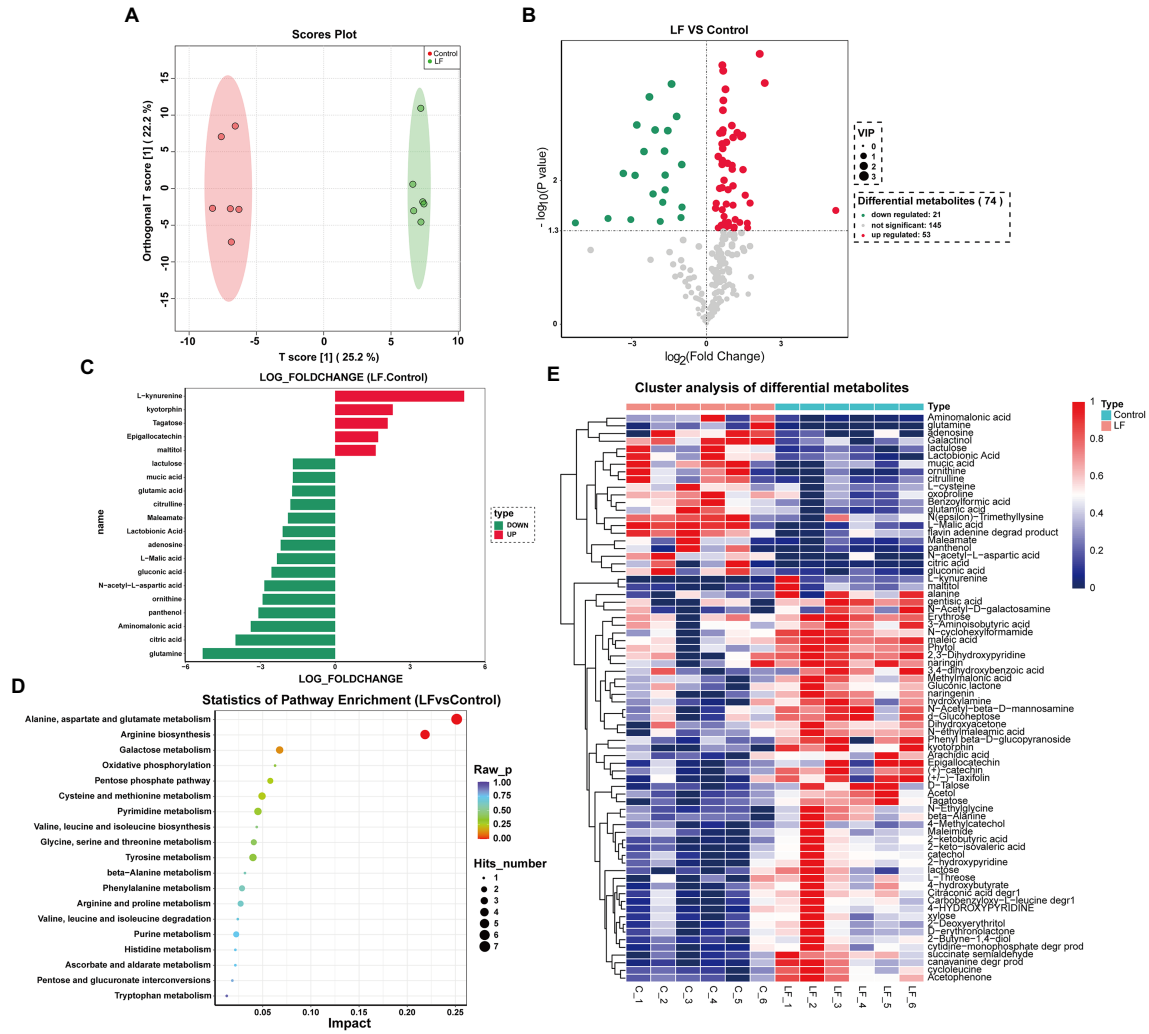
Cluster analysis of differential metabolites and KEGG enrichment analysis. **(A)** OPLS-DA diagram of comparison combination; **(B)** volcanic map of differential metabolites screening between combinations; **(C)** column diagram of differential metabolites between combinations; **(D)** KEGG metabolic pathway analysis of each comparison combination; **(E)** hierarchical cluster analysis heat map of each comparison combination.

We also conducted multivariate statistical analyses using orthogonal partial least squares discriminant analysis (OPLS-DA) (Boccard and Rutledge, 2013). The OPLS-DA score chart showed high significance in the two groups’ scores, while all samples were in the 95% confidence interval (Figure 3A). Therefore, these samples could be used for subsequent differential metabolite analysis.

#### 3.4.2. Differential metabolite analysis

As depicted in Figure 3B, 74 differential metabolites were identified between the LF and Control groups, including 53 up-regulated and 21 down-regulated metabolites. Statistical analysis of the top 20 differential metabolites demonstrated that OsiLf-2 addition could affect metabolite composition and classification in roots, such as the up-expression of phenolic compounds (kyotorphin, Epigallocatechin), amino acid (L−kynurenine), polysaccharide (Tagatose) and maltitol; and the down-expression of organic acids (mucic acid, Lactobionic acid, L−Malic acid, citric acid, gluconic acid, Aminomalonic acid), vitamin (panthenol), amino acids (glutamic acid, citrulline, ornithine, N−acetyl−L−aspartic acid, adenosine), amines (maleamate, glutamine) and sugar (lactulose) (Figure 3C). These data suggest that OsiLf-2 can induce *Camellia oleifera* roots to produce more sugars and phenolic substances to help the plant resist drought stress for maintaining normal growth. Moreover, OsiLf-2 could cause *C. oleifera* to reduce small molecule secretion, such as organic and amino acids, decreasing plant energy consumption to resist drought stress further.

#### 3.4.3. Differential metabolite clustering and KEGG enrichment analyses

A metabolite’s relative content can be reflected by its abundance. Therefore, hierarchical clustering analysis of differential metabolites helped group metabolites with the same characteristics into one category and identified characteristic changes among experimental groups. These results signified that all differential metabolites between the LF and Control groups could be divided into two clusters (Figure 3E). The first cluster included 21 metabolites, primarily organic and amino acids, whose relative expression levels were higher in the Control group than in the LF group. In contrast, the second cluster contained 53 metabolites, mainly polysaccharides, sugar alcohols, and amines, whose relative expression levels were higher in the LF group than in the control group.

To elucidate the biological functions of the differential metabolites between the paired groups, comparison searches and metabolic pathway analyses were carried out on these metabolites based on the KEGG database. As shown in Table 3, the metabolic pathway analysis identified 52 metabolic pathways. KEGG enrichment analysis revealed that differential metabolites were enriched in 19 pathways, among which several pathways with the most enriched differential metabolites and significant differences involved amino acid and polysaccharide biosynthesis and metabolism, as well as the pentose phosphate pathway (Figure 3D). They are pathways of Alanine, Aspartate and glutamate metabolism, Arginine biosynthesis, Galactose metabolism, Pentose phosphate pathway, Cysteine and methionine metabolism, Pyrimidine metabolism, Glycine, Serine and threonine metabolism, Tyrosine metabolism. These results indicated that OsiLf-2 could induce plant production of additional small osmotic substances such as amino acids and polysaccharides, subsequently increasing a plant root’s osmotic pressure for protection from drought stress damage.

**Table 3:** Metabolic pathway analysis.

Pathway	Total	Hits	Raw_p	FDR
Citrate cycle (TCA cycle)	20	3	0.0165	0.1394
Pentose phosphate pathway	35	2	0.2543	0.601
Pentose and glucuronate interconversions	55	1	0.7937	0.8973
Galactose metabolism	45	3	0.1272	0.4596
Ascorbate and aldarate metabolism	47	1	0.7396	0.8741
Oxidative phosphorylation	16	1	0.3647	0.7294
Arginine biosynthesis	23	5	0.0003096	0.008049
Purine metabolism	92	2	0.7379	0.8741
Pyrimidine metabolism	68	3	0.2929	0.6347
Alanine, aspartate and glutamate metabolism	28	7	6.173e-06	0.000321
Glycine, serine and threonine metabolism	50	2	0.4091	0.7781
Cysteine and methionine metabolism	62	3	0.2468	0.601
Valine, leucine and isoleucine degradation	42	1	0.699	0.8741
Valine, leucine and isoleucine biosynthesis	23	1	0.4798	0.8464
Arginine and proline metabolism	76	2	0.6348	0.8464
Histidine metabolism	47	1	0.7396	0.8741
Tyrosine metabolism	77	3	0.3631	0.7294
Phenylalanine metabolism	72	2	0.6048	0.8464
Tryptophan metabolism	81	1	0.9041	0.9594
beta-Alanine metabolism	32	1	0.5982	0.8464
Taurine and hypotaurine metabolism	22	3	0.02145	0.1394
Selenocompound metabolism	27	1	0.5361	0.8464
Cyanoamino acid metabolism	45	1	0.724	0.8741
Glutathione metabolism	38	4	0.0194	0.1394
Starch and sucrose metabolism	51	2	0.419	0.7781
Amino sugar and nucleotide sugar metabolism	108	2	0.8157	0.9025
Glycerolipid metabolism	34	1	0.6207	0.8464
Pyruvate metabolism	31	2	0.2126	0.5796
Glyoxylate and dicarboxylate metabolism	60	4	0.08229	0.389
Propanoate metabolism	48	3	0.1465	0.4762
Butanoate metabolism	41	3	0.1031	0.4467
C5-Branched dibasic acid metabolism	32	2	0.2229	0.5796
Carbon fixation in photosynthetic organisms	23	2	0.1326	0.4596
Thiamine metabolism	30	1	0.5744	0.8464
Vitamin B6 metabolism	28	1	0.5493	0.8464
Nicotinate and nicotinamide metabolism	55	3	0.195	0.5632
Pantothenate and CoA biosynthesis	28	2	0.1818	0.5561
Porphyrin and chlorophyll metabolism	124	1	0.9737	1
Nitrogen metabolism	19	3	0.0143	0.1394
Sulfur metabolism	32	1	0.5982	0.8464
Flavonoid biosynthesis	68	3	0.2929	0.6347
Isoflavonoid biosynthesis	63	1	0.8368	0.9065
Aminoacyl-tRNA biosynthesis	52	4	0.05363	0.2789
Biosynthesis of unsaturated fatty acids	54	1	0.7876	0.8973
Metabolic pathways	1,653	25	1	1
Biosynthesis of secondary metabolites	1,024	13	1	1
Carbon metabolism	112	10	0.0006979	0.0121
2-Oxocarboxylic acid metabolism	134	4	0.5203	0.8464
Biosynthesis of amino acids	127	8	0.02106	0.1394
Vancomycin resistance	24	1	0.4945	0.8464
ABC transporters	126	6	0.1323	0.4596
Sulfur relay system	10	2	0.02958	0.1709

Pathway, metabolic pathway; Total, the number of metabolites in the metabolic pathway; Hits, number of differential metabolites hit the pathway; Raw_p, *P*-value of metabolic pathway enrichment analysis; FDR, *P*-value corrected by multiple hypothesis testing.

## 4. Discussion

### 4.1. OsiLf-2 assists *Camellia oleifera* in resisting drought stress and improves fruit quality

Water shortage and drought in summer and autumn are one of the prominent factors limiting *Camellia oleifera* yield and quality. Drought reduces yield, decreases fruit oil content, and even causes death in drought-sensitive varieties (Dong et al., 2017). Indeed, this study also found that *C. oleifera* fruit traits indexes, such as fruit and seed heights, diameters, and weights, were reduced under drought conditions compared to the blank control group (Table 1). We also observed that drought severely reduced oil quality traits such as oil content and stearic, oleic, and linolenic acids (Table 2).

Utilizing microorganisms with stress tolerance to overcome drought problems is a viable strategy, as these microorganisms play a crucial role in maintaining plant growth performance under stressful conditions (Papworth et al., 2014). Actinobacteria is necessary microbial resources and can alleviate the adverse effects of drought stress and increase crop yield (Selim et al., 2019). *Streptomyces*, a common actinobacteria genus, exhibits remarkable performances in salt and drought tolerance and can improve plant’s tolerance under abiotic stress conditions (Sadeghi et al., 2012; Abbasi et al., 2020). We previously isolated an endophytic *Streptomyces albidoflavus* OsiLf-2, which enhanced host rice resistance to drought and salt stresses. In this study, we investigated whether OsiLf-2 application could improve the drought resistance of *C. oleifera*.

Our results showed that OsiLf-2 application to *C. oleifera* after drought treatment significantly improved fruits and seeds traits such as fruit diameter and weight, which almost reached the same level as those of the blank control (CK) group (Table 1). Moreover, OsiLf-2 treatment significantly increased oil content in kernel, palmitic and linoleic acid contents (Table 2). These observations indicate that exogenous OsiLf-2 can improve *C. oleifera* fruit and seed quality, thus alleviating the adverse effects from drought stress. This study agrees with the study of AbdElgawad et al. (2019) in which actinobacteria isolated from *Phoenix dactylifera* L. roots and used as an inoculant in a semi-arid environment improved soil fertility and further increased date palm fruit yield.

### 4.2. OsiLf-2 affects the bacterial community structure in *Camellia oleifera* rhizosphere soil

Beneficial microorganisms can assist bacteria in forming biofilms by producing osmolyte exopolysaccharides (EPS) to create a microenvironment that retains moisture and water levels. Moreover, they participate in attaching bacteria to root and soil surfaces. All these activities help facilitate plant-microbe interactions and protect microbes and plant roots from drought stress (Nadeem et al., 2021). For instance, Niu et al. (2022) found that OsiLf-2 can produce large EPS and biofilm quantities in rice roots under drought conditions to protect the host from adverse drought and water shortage effects. In the present study, soil bacterial diversity analysis revealed that compared to the control group, the soil bacterial community diversity and abundance decreased after OsiLf-2 application to *C. oleifera* following drought treatment (Figure 1C). This may be explained by the fact that OsiLf-2 application to the roots of *C. oleifera* leads to production of a large amount of EPS; this subsequently wrap the roots to reduce excessive water loss, alleviate drought damage, and protect normal plant growth. Similarly, Sandhya et al. found that sunflower seedlings treated with EPS-producing *Pseudomonas putida* resulted in biofilm formation on the plant root surface, which increased stable soil aggregate percentage to better protect plants against drought stress (Sandhya et al., 2009).

While organic acids are significant components of plant root exudates and participate in essential nutrient dissolution and release (e.g., phosphorus), these nutrients also serve as soil microbes’ primary energy source and key rhizobacterial chemotaxis drivers (Macias-Benitez et al., 2020). In addition, amino acids are essential carbon and nitrogen sources for soil microbes, which can regulate microbial community structures and enhance soil nutrient cycling (Feng et al., 2021). In this study, transcriptome and metabolome analyses revealed that organic and amino acid synthesis decreased in *C. oleifera* roots. KEGG enrichment analysis showed that four down-regulated DEGs between the LF and Control groups were annotated into cyanoamino acid metabolism (Figure 2E). Likewise, metabolome analysis found that OsiLf-2 application to *C. oleifera* after drought treatment down-regulated six organic and five amino acid expressions (Figure 3C). Differential metabolites’ hierarchical clustering analyses showed that organic and amino acid relative expression levels were lower in the LF group with OsiLf-2 application than in the drought treatment control group (Figure 3E). These observations indicated that OsiLf-2 might produce EPS to wrap *C. oleifera* roots to hinder the release of other small molecule root exudates. This subsequently affects chemotaxis enrichment and rhizosphere soil bacterial community composition, therefore decreasing the diversity and abundance of microbes. These results are also consistent with the analysis of bacterial diversity in our rhizosphere soil (Figure 1C).

### 4.3. OsiLf-2 induces osmolyte synthesis in *Camellia oleifera* roots

Osmolytes include a variety of inorganic ions and organic solutes that decrease osmotic stress by increasing concentrations to improve cellular water retention during drought stress (Fang and Xiong, 2015). Among them, rising sugar and sugar alcohol concentrations can significantly reduce cellular osmotic stress, exerting an effect on the osmoregulatory process under drought stress (Granda and Camarero, 2017). In the present study, GO enrichment analysis consistently showed that among the up-regulated DEGs between the LF and Control groups, three (Novel06804, Novel06807, and Novel06560) were annotated into BP’s glycosyl compound metabolic process, three (Novel11368, Novel11367, and Novel03250) were annotated into polysaccharide binding, and two (Novel11367, Novel11368) were annotated into MF’s starch binding (Figure 2B). Differential metabolite statistical analysis also revealed that OsiLf-2 application to *C. oleifera* after drought treatment up-regulated tagatose, maltitol, two polysaccharides, and sugar alcohol expressions (Figure 3C). Moreover, differential metabolite hierarchical clustering analysis found that sugar and sugar alcohol relative expression levels were higher in the LF group than in the Control group (Figure 3E).

Sugars and sugar alcohols interact with various enzymes and membrane protein complexes to prevent water loss, alleviate damage, and protect cellular structures (Li et al., 2011). Richardson et al. (1992) also discovered that endophytic bacterial infection of tall fescue resulted in various sugar alcohol and carbohydrate accumulation in plant leaves, which improved plant drought tolerance, osmotic potential, and regeneration capacity. These data further demonstrated that OsiLf-2 might induce the synthesis of sugars and sugar alcohols osmolytes in *C. oleifera* roots to protect plant cells from drought stress, thereby maintaining their normal physiological activities. Similarly, some studies have also shown that the content of sugars and total phenols in plant roots increased under drought stress (Gao et al., 2020), which is consistent with our results (Figure 3C).

### 4.4. OsiLf-2 induces *Camellia oleifera* antioxidant system activation to resist drought stress

Plants accumulate large amounts of reactive oxygen species (ROS) under unfavorable conditions such as drought, which seriously affects plant growth and development. The antioxidant system can scavenge ROS generated under drought conditions, thereby mitigating oxidative damage (Fei et al., 2020). This system mainly includes ascorbic acid peroxidase, catalase, glutathione reductase, and superoxide dismutase, while it accumulates several non-enzymatic antioxidants, such as ascorbic acid, cysteine, and glutathione (del Rio et al., 2003). This study showed that OsiLf-2 could induce *C. oleifera* antioxidant system activation to resist drought stress. GO enrichment analysis revealed that five up-regulated DEGs between the LF and Control groups (Novel13433, Novel13432, Novel11181, Novel02124, and Novel11024) were annotated into MF’s peroxidase activity (Figure 2B).

Moreover, differential metabolite clustering analysis between the LF and Control groups found that antioxidant-cysteine’s relative abundance was higher in the LF group than in the Control group (Figure 3E). These differential metabolites were also annotated into cysteine and methionine metabolic pathways based on the KEGG enrichment analysis (Figure 3D). These results indicated that OsiLf-2 could induce the host plant to increase its peroxidase activity and synthesize antioxidants such as cysteine, thus activating the antioxidant system to resist drought stress.

## 5. Conclusion

The endophytic *Streptomyces albidoflavus* OsiLf-2 can assist *Camellia oleifera* in alleviating drought stress and improving fruit quality. This study used a multi-omics joint analysis of microbiomes, transcriptomes, and metabolomes to reveal that OsiLf-2 might induce the syntheses of osmolytes in *C. oleifera* roots, such as polysaccharides and sugar alcohols, thus alleviating the adverse effects on root cells under drought stress. OsiLf-2 might also induce *C. oleifera* to increase peroxidase activity and synthesize antioxidants such as cysteine, thus activating the plant antioxidant system to protect plant cells against drought stress for normal growth. Thus, the application of endophytic actinobacteria provides a prospective biotechnological approach for developing stress-resistant plants.

## Data availability statement

The datasets presented in this study can be found in online repositories. The names of the repository/repositories and accession number(s) can be found at: https://www.ncbi.nlm.nih.gov/, PRJNA935928.

## Author contributions

ZH and KC: software, and writing – review and editing. YZ: validation. RW and XW: investigation. ZZ and TX: data curation. ZH and KC: writing – original draft preparation. YC and YZ: supervision and project administration. YC: funding acquisition. All authors have read and agreed to the published version of the manuscript.

## Funding

This research was funded by the Seed Industry Innovation Project of Hunan Province (2021NK1007), the Major special project of Changsha Science and Technology Bureau (KQ2102007), and the Natural Science Foundation of Hunan Province (Grant No. 2022JJ30325).

## Conflict of interest

The authors declare that the research was conducted in the absence of any commercial or financial relationships that could be construed as a potential conflict of interest.

## Publisher’s note

All claims expressed in this article are solely those of the authors and do not necessarily represent those of their affiliated organizations, or those of the publisher, the editors and the reviewers. Any product that may be evaluated in this article, or claim that may be made by its manufacturer, is not guaranteed or endorsed by the publisher.

## Footnotes

^1^ http://www.kegg.jp

^2^ http://www.metaboanalyst.ca/
