# Supplementary material for: Multi-omics joint analysis reveals how Streptomyces albidoflavus OsiLf-2 assists Camellia oleifera to resist drought stress and improve fruit quality
Source: Front Microbiol. 2023 Mar 17;14:1152632. doi: 10.3389/fmicb.2023.1152632 (PMC10063849; doi:10.3389/fmicb.2023.1152632)
Supplement: Supplementary file 1 [file Table_1.DOCX]

**Supplementary material**

**Table S1** Transcriptome sequencing data statistics

| Sample | Raw Reads | Raw Bases | Clean Reads | Clean Bases | Error Rate | Q20 | Q30 | GC Content |
| --- | --- | --- | --- | --- | --- | --- | --- | --- |
| C_1 | 46868244 | 7.03G | 46163370 | 6.92G | 0.02% | 97.94% | 94.34% | 45.23% |
| C_2 | 43841602 | 6.57G | 43095160 | 6.46G | 0.03% | 97.89% | 94.32% | 45.67% |
| C_3 | 40114596 | 6.01G | 39297060 | 5.89G | 0.02% | 97.93% | 94.43% | 45.80% |
| LF_1 | 40345412 | 6.05G | 39296310 | 5.89G | 0.03% | 97.90% | 94.36% | 46.09% |
| LF_2 | 46933962 | 7.04G | 45625840 | 6.84G | 0.03% | 97.92% | 94.29% | 45.51% |
| LF_3 | 40697898 | 6.1G | 39698536 | 5.95G | 0.02% | 97.94% | 94.40% | 45.70% |

Raw reads and Raw bases: the number of Reads and the total base amount ( in G ) of the original sequencing data; Clean reads and Clean bases: the number of Reads and the amount of bases ( in G ) obtained after quality control; Q20 and Q30: the proportion of bases with Phred mass value greater than 20 and 30 in the total bases; GC content: The ratio of GC to total bases in sequencing data.

**Table S2** Comparison of Reads with reference sequences

| Sample name | C_1 | C_2 | C_3 | LF_1 | LF_2 | LF_3 |
| --- | --- | --- | --- | --- | --- | --- |
| Total reads | 46163370 | 43095160 | 39297060 | 39296310 | 45625840 | 39698536 |
| Total mapped | 40785882 (88.35%) | 37649538 (87.36%) | 33937176 (86.36%) | 33947904 (86.39%) | 40584326 (88.95%) | 35224204 (88.73%) |
| Multiple mapped | 4910842 (10.64%) | 4708954 (10.93%) | 4289520 (10.92%) | 4288992 (10.91%) | 5122032 (11.23%) | 4550750 (11.46%) |
| Uniquely mapped | 35875040 (77.71%) | 32940584 (76.44%) | 29647656 (75.44%) | 29658912 (75.48%) | 35462294 (77.72%) | 30673454 (77.27%) |
| Read-1 | 17937520 (38.86%) | 16470292 (38.22%) | 14823828 (37.72%) | 14829456 (37.74%) | 17731147 (38.86%) | 15336727 (38.63%) |
| Read-2 | 17937520 (38.86%) | 16470292 (38.22%) | 14823828 (37.72%) | 14829456 (37.74%) | 17731147 (38.86%) | 15336727 (38.63%) |
| Reads map to '+' | 17937520 (38.86%) | 16470292 (38.22%) | 14823828 (37.72%) | 14829456 (37.74%) | 17731147 (38.86%) | 15336727 (38.63%) |
| Reads map to '-' | 17937520 (38.86%) | 16470292 (38.22%) | 14823828 (37.72%) | 14829456 (37.74%) | 17731147 (38.86%) | 15336727 (38.63%) |
| Non-splice reads | 22772881 (49.33%) | 20599894 (47.8%) | 18113647 (46.09%) | 18810092 (47.87%) | 22602202 (49.54%) | 19367790 (48.79%) |
| Splice reads | 13102159 (28.38%) | 12340690 (28.64%) | 11534009 (29.35%) | 10848820 (27.61%) | 12860092 (28.19%) | 11305664 (28.48%) |

Total reads: the number of sequencing sequences filtered by sequencing data ( Clean data ); Total mapped: statistics of the number of sequenced sequences that can be mapped to the genome; Multiple mapped: the number of sequencing sequences with multiple alignment positions on the reference sequence; Uniquely mapped : the number of sequencing sequences with unique alignment position on the reference sequence; Read-1, Read-2: read1, read2 were compared on the number of sequences statistics; Reads map to ' + ', Reads map to ' - ': sequencing sequence alignment to the statistics of positive and negative strands on the genome; Non-splice reads: the statistics of sequencing sequences aligned to the whole exon; Splice reads: in the statistics of sequencing sequences ( also known as Junction reads ) aligned to two exons, the percentage of Splice reads depends on the length of the sequenced fragment.

**Table S3** Ion number statistics

| Mode | Total ion number | After pre-processing | Ratio(%) |
| --- | --- | --- | --- |
| GC | 245 | 226 | 92.24% |
